# Phytochemical characterization and antimicrobial activity of *Nigella sativa* seeds

**DOI:** 10.1371/journal.pone.0272457

**Published:** 2022-08-04

**Authors:** Festus S. Shafodino, Julien M. Lusilao, Lamech M. Mwapagha

**Affiliations:** Department of Natural and Applied Sciences, Faculty of Health and Applied Sciences, Namibia University of Science and Technology, Windhoek, Namibia; Universidad Autonoma de Chihuahua, MEXICO

## Abstract

*Nigella sativa* is one of the medicinal plant species that gained popularity for a wide range of medicinal applications due to its seeds which are rich in phytoconstituents. Continuous scientific investigations on *N*. *sativa* seeds are needed to better understand its many medicinal potentials. This will also form a composition-based foundation that support several old and/or new case beneficial histories of its seeds. In this study, the antimicrobial activity of *N*. *sativa* seeds was phytochemically characterized and evaluated. Different extracts of *N*. *sativa* seeds were obtained by maceration and soxhlet extraction methods using different extraction solvents. The obtained extracts were tested using UV-Vis, FTIR, TLC, and GC-MS techniques. Antimicrobial analysis against pathogenic bacterial strains (*E*. *coli*, *P*. *aeruginosa*, *S*. *aureus* and *B*. *subtilis*) was carried out by disc diffusion method using different preparations of *N*. *sativa* seeds. The screening analysis revealed the presence of all the tested phytochemicals. FT-IR analysis of *N*. *sativa* seeds oil extracted with absolute ethanol revealed functional groups that are associated with active ingredients of medicinal value. The GC-MS chromatograms revealed different chemical constituents whose known bioactivities and/or applications are essential in the management of life-threatening infections. Different extracts of *N*. *sativa* seeds showed antimicrobial activity with different efficacy against the tested pathogenic bacterial strains. Therefore, this study shows that extracts of *N*. *sativa* seeds contain a variety of chemical components and functional groups linked to their antimicrobial properties, and they might be natural precursors of nutraceuticals.

## Introduction

The emerging number of bacterial infections and antibiotic resistance have become a major threat in most parts of the world. Modern medical treatments including organ transplantations, chemotherapy, and surgeries might become risky without the use of effective antibiotics. Recently, the exploration of plants for therapeutic purposes gained popularity due to several reasons, such as, easy access without prescription, low-cost, natural origin, and possibility of reducing the need for synthetic drugs that may have severe side effects [1–3]. Medicinal plants have always been the critical natural factories of phytochemicals such as flavonoids, tannins, phenols, steroids, alkaloids and terpenoids which are responsible for their biological activities [3]. Plant products that are generated from fruits, flowers, seeds, roots, leaves, and barks are part of phytomedicines and many bioactive constituents of plants have been detected and further characterized by using different standard analytical methods [3, 4].

*Nigella sativa* is one of the medicinal plant species that gained popularity for a wide range of medicinal applications due to its seeds, generally known as black seeds, which are rich in phytoconstituents. Series of studies reported that thymoquinone is the major constituent of *N*. *sativa* seeds and accounts for most of the seeds’ pharmacological properties [5–8]. Literature also indicates that extracts and/or oil of *N*. *sativa* seeds contain many other constituents such as proteins, carbohydrates, vitamins, dietary minerals (such as Fe and Zn), crude fiber, alkaloids, saponins, steroid, terpenoids, p-cymene, limonene, and fatty acids [3, 7, 9].

The reported pharmacological properties that are imparted by *N*. *sativa* seeds due to their chemical composition include but not limited to, analgesic, appetizer, antidiabetic, antioxidant, anti-inflammatory, radical scavenger, and antimicrobial properties [3, 4, 9]. Although the phytochemical composition and pharmacological properties of *N*. *sativa* seeds have been widely explored, Dinagaran, Sridhar and Eganathan [10] reported that the complete chemical profile of *N*. *sativa* seeds oil (NSSO) and/or extracts is not yet determined. In this view, it is necessary to re-evaluate the therapeutic properties, such as the promising antimicrobial effects, of herbal extracts and/or oils due to the escalating search of natural products that confer desirable antibacterial or antimicrobial properties against antibiotic resistance [11] as well as to provide the up-to-date knowledge on their compositional profile and ascertain their medicinal properties.

On another hand, the basis of many applications such as raw and processed food preservation, pharmaceuticals, and natural therapies is formed by the antimicrobial activities of extracts derived from medicinal plants [2, 10, 12–14]. Investigations pertaining to the biochemical analysis to identify phytochemical constituents and antimicrobial properties of natural products such as extracts and/or oil *N*. *sativa* seeds offer new opportunities to discover and formulate effective antibiotics as alternative treatments in the case of drug resistant pathogenic bacterial strains [13, 15, 16]. This study therefore aimed to investigate the chemical composition of the extracts and/or oil of *N*. *sativa* seeds and to infer from these results the phytoconstituents that are responsible for their corresponding antimicrobial activity against various pathogenic bacterial strains as well as to compare these finding with those reported worldwide.

## Materials and methods

### Extraction of oil from *N*. *sativa* seeds

*N*. *sativa* seeds were purchased from the local market in Windhoek (Namibia), washed and allowed to dry at room temperature before grinded into a fine powder. The seeds powder was used for the preparation of different medicinally active extracts and oil.

The oil was extracted from 25.0 g of *N*. *sativa* seeds powder in a Soxhlet apparatus using 250 mL of two different solvents, namely absolute ethanol (99.9% v/v) and hexane (Skylabs’ chemicals) at room temperature for 4 hours and 2 hours, respectively [9, 10]. The hexane oil was concentrated with the rotary evaporator by evaporating the extractants at 40–50°C under reduced pressure whereas the ethanol oil extract was separated to the crude extract by decantation with a separatory funnel. The oils and ethanol crude extract were then stored at 4°C until further use.

### Preparation of different solvent extracts of *N*. *sativa* seeds by maceration extraction method

The organic solvent extracts were prepared using the sequential maceration method with extractants of increasing polarity, namely petroleum spirit (Skychem), ethyl acetate (Skychem) and methanol (Skylabs) as outlined by Mengesha Yessuf [3] with slight modifications. About 20 g of *N*. *sativa* seed powder were macerated with 100 ml of each solvent for 24 hours at room temperature with agitation at 150 rpm and the desired filtrates were separated from the solid residues using the Whatman No. 1.5 filter papers. All filtrates were pre-concentrated under reduced pressure using the rotary evaporator at 40–60°C to yield the crude extracts (petroleum spirit extract, ethyl acetate extract and methanol extract, respectively). The aqueous extract was also prepared with deionized water using the same procedure as above. All the extracts were stored at 4°C until analysis.

### Qualitative phytochemical screening

The obtained extracts were subjected to phytochemical screening using standard methods [3, 17, 18] that are summarized below. The general reactions in this assay signaled the presence or absence of major phytochemical phytoconstituents in the extracts of *N*. *sativa* seeds.

#### Test for alkaloids (Wagner’s reagents)

An amount of 1.5 ml of 1% hydrochloric acid (HCl) was added to 2.0 ml of each extract in a test tube. 6 drops of Wagner’s reagent were added after heating the test tube content over the water bath. The presence of alkaloids was showed by the formation of an orange precipitate.

#### Test for flavonoids

A few drops of ferric chloride hexahydrate (FeCl_3_∙6H_2_O) solution were added to 2.0 ml of each extract. The formation of an intense green color indicates the presence of flavonoid.

#### Test for phenols

A few drops of 5% FeCl_3_∙6H_2_O solution were added to 2.0 ml of each extract. The presence of tannins was showed by a deep blue-black color.

#### Test for tannins

One ml of each extract was mixed with 2.0 ml of distilled water. To this mixture, 2.0 ml of 5% FeCl_3_∙6H_2_O solution and the resulting brownish-green or dark-green solution confirms the presence of tannin.

#### Test for cardiac glycosides

Three mL of glacial acetic acid (CH_3_COOH) were added to 2.0 ml of each extract in the test tube followed by addition of 1 drop of 5% FeCl_3_∙6H_2_O. 0.5 mL of concentrated sulphuric acid (H_2_SO_4_) was added carefully by the side of the test tube. The blue color was formed in CH_3_COOH indicating the presence of Cardiac glycosides.

#### Test for steroids

Five ml of chloroform (CHCl_3_) and 2.0 ml acetic anhydride ((CH₃CO)₂O) were added to 2.0 ml of each extract followed by concentrated H_2_SO_4_. The reddish-brown coloration at the interface shows the presence of steroids.

#### Test for saponins

Each extract was diluted with distilled water and shaken in a test tube for 15 minutes. The presence of saponins is indicated by the formation of a layer of foam.

#### Test for terpenoids

Two ml of CHCl_3_ were mixed with 1.0 ml of extract and 3.0 ml concentrated H_2_SO_4_ were carefully added to form a layer. The presence of terpenoids was indicated by a reddish-brown coloration at the interface.

### Quantitative analysis of phytochemicals in aqueous and methanol extracts

#### Determination of Total Tannin Contents (TTC)

The standard tannic acid solution was prepared by dissolving 50 mg of tannic acid (LD Didactic) in 250 ml of distilled water. Serial dilution was peformed using distilled water to prepare different concentrations (0,10, 20, 40, 60, 80 and 100 mg/L) of tannic acid standard in 50 ml volumetric flasks for the standard curve of tannic acid. The TTC was determined using Van-Burden and Robinson method [19, 20]. Five ml of each standard (0, 10, 20, 40, 60, 80 and 100 mg/L of tannic acid) and one ml of each extract were added to different test tubes. The content of each test tube was mixed with 2 ml of 0.1 M FeCl_3_∙6H_2_O (LD Didactic) followed by 4 drops of 0.1 N HCl (Promark) and 3 drops of 8 mM C_6_FeK_4_N_6_ (LD Didactic). Distilled Water was used as a blank. The absorbance was measured at 760 nm in triplicates within 10 minutes using the Lambda 365 UV-Vis spectrophotometer (PerkinElmer). The graph of absorbance versus the concentrations of tannic acid was plotted. The content of tannin in each sample was determined by using the linear equation from the graph as μg of tannic acid equivalents (TAE)/g of powdered seed [21].

#### Determination of Total Phenolic Contents (TPC)

The TPC was determined in both methanol and aqueous extract of *N*. *sativa* seeds using a method outlined by Patle et al. [22] with slight modifications. Two mL of 10% Folin-Ciocalteu reagent (Merck) were added to 1 ml of both the standards solution of gallic acid (Merck) (10 mg/ml– 100 mg/ml) and extracts in separate test tubes, and 4 ml of Na_2_CO_3_ (LD Didactic) were added after 5 minutes. Thereafter, 2 ml of methanol (Promark) were added to each mixture in the test tube and topped to 10 ml with distilled water. Each test-tube content was well shaken and incubated at room temperature for 30 minutes. The absorbance was measured at 765 nm in triplicates using the Lambda 365 UV-Vis spectrophotometer (PerkinElmer) against methanol as a blank. The total phenolic content was calculated from the standard calibration curve for gallic acid and expressed as mg gallic acid equivalents (GAE)/ g of powdered seed.

#### Determination of Total Flavonoid Contents (TFC)

The TFC in both methanol and aqueous extract of *N*. *sativa* seeds was determined by the AlCl_3_ colorimetric method [23, 24] using the Lambda 365 UV-Vis spectrophotometer (PerkinElmer). An amount of 2 ml of quercetin (Sigma) standard solutions (range: 0.5 ug/ml– 15 μg/ml) of quercetin or 1 ml of each extract was mixed separately with 2 ml of 2% AlCl_3_ (Sigma) followed by 2 ml of 120 mM of CH_3_COOK (Merck) and 4 ml of distilled water. The choice of the calibration range was due to the fact that a deviation from linearity was observed at higher concentration of quercetin. These solutions were then incubated at room temperature for 1 hour. The absorbance was measured at 425 nm against methanol as a blank and a blank reagent composed of distilled water instead of the sample/standard [23]. The content of total flavonoid in the extracts was calculated from quercetin calibration curve (y = mx + c, x = (y-c)/m) and expressed as mg of quercetin equivalents (QE)/g of dried plant material. The determinations were performed in triplicate.

### Characterization of the extracts of *N*. *sativa* seeds

#### Thin Layer Chromatography (TLC) profiling of methanol extract of *N*. *sativa* seeds

Methanol is known to be effective in extracting maximum number of medicinal constituents of interest than water and other organic solvents (petroleum spirit and ethyl acetate) to a considerate extend. Hence, only the methanol extract of *N*. *sativa* seeds was subjected to TLC profiling using the method outlined by Mengesha Yessuf [3] with minor modifications. The extract was spotted manually on two TLC plates using a capillary tube and they were exposed to the mobile phases made of different solvent ratios petroleum spirit/ethyl acetate/methanol (2:3:5 and 6:2:2). Spots were visualized using the UV lamp and the presence of spots with trailing was revealed. This was done in triplicate on each plate for comparison.

#### Gas Chromatography-Mass Spectrometry (GC-MS) analysis

The chromatographic analyses of both methanol extract and hexane oil extract of *N*. *sativa* seeds were carried out with the clarus® 680 (Perkin Elmer) GC-MS instrument under computer control at 70 eV as per previously reported methods [9, 11]. These extracts were chosen for GC-MS analysis due to their volatility. The compounds in the extracts of interest were identified by comparing their mass spectra and retention indices to those found in the National Institute of standards and Technology (NIST) library.

#### The Fourier Transform Infrared (FT-IR) analysis

The more viscous extract (i.e. the one obtained using absolute ethanol) was analysed using the Spectrum Two FT-IR (Perkin Elmer) for the identification of the different functional groups (antibacterial features) present in *N*. *sativa* seeds oil (NSSO).

### Antimicrobial activity of the extracts of *N*. *sativa* seeds

The disk diffusion method was used to investigate the antimicrobial activity of *N*. *sativa* seeds extracts against pathogenic bacterial strains (*Escherichia coli*, *Pseudomonas aeruginosa*, *Staphylococcus aureus* and *Bacillus subtilis*). The bacterial strains cultures were acquired from the Namibia Institute of Pathology (NIP) and subcultured onto the flesh nutrient agar [11]. Five colonies were picked from the fresh nutrient agar culture to prepare the bacteria suspension of each strain to be tested and inoculated in 3 ml of distilled water. After five minutes, 100 μL of each strain suspension was spread onto the plates of nutrient agar. Paper discs (5 mm) prepared from Whatman no. 1.5 filter papers were placed on top of the innoculated plates. The extracts or preparations of *N*. *sativa* seeds, oil extracted with hexane, oil extracted with absolute ethanol, methanol extract and aqueous extract (undiluted and diluted 1:1 in ethylene glycol (LD Didactic) were tested for their inhibitory activity against the pathogenic bacteria by pippeting 10 *μ*L of each preparation or extract onto the 5 mm paper discs. The volume of each preparation delivered onto each disc was selected on the basis that plants may exhibit antimicrobial properties when assayed at an adequate amount [17, 25]. Distilled Water was used as a negative control in addition to the extracts while 5 mm discs of a known antibiotic (Streptomycin) was selected among others and used as a positive control for comparison. All plates were incubated at 37°C for 18–24 hours. The zones of inhibition were observed, measured using the digital vernier caliper including the 5 mm sized discs, and recorded. This was done in triplicate.

## Results and discussion

### Extraction yield of oil from *N*. *sativa* seeds

The colour of the oil obtained ranged from light brown to brown which is similar to the desirable brownish yellow colour from a previously reported study on the physicochemical characteristics of *N*. *sativa* seeds oil [26]. The maceration extraction was also preferred to prepare extracts from *N*. *sativa* seeds due to its convenience and cost-effective way of obtaining thermolabile drugs [27, 28]. In this study, although absolute ethanol extracted more active components from *N*. *sativa* seeds than hexane, the latter produced a higher extract yield (60%) than ethanol (16%). These findings are in conformity with those reported by Padalia and Chanda [15] in which hexane had more extractive yield than acetone (a polar solvent) and it was concluded that non-polar compounds may be more efficient in extracting *N*. *sativa* oil than the polar compounds. It is important to select the best extraction method and appropriate extraction solvents in relation to the intended use of the final medicinal plant extract (such as identification or separation of bioactive compounds and test for biological activities) since they have influence on the extraction yield [15]. However, the extractive yield is not a measure of biological activities and thus, higher yield of NSS oil extracted using hexane does not imply a higher antimicrobial activity than NSS oil extracted with absolute ethanol.

Furthermore, the oil extracted using absolute ethanol was greasy and viscous. This was also observed by Wong et al. [29] with the essential oil extracted from Cinnamon using ethanol which was associated with high viscosity. This may be accountable for the differences in extraction yield of the oil obtained in the current study in addition to the extraction time, temperature, and the strength of the types of solvents used [29, 30].

### Qualitative screening of phytochemicals from different extracts of *N*. *sativa* seeds

**Fig 1:**
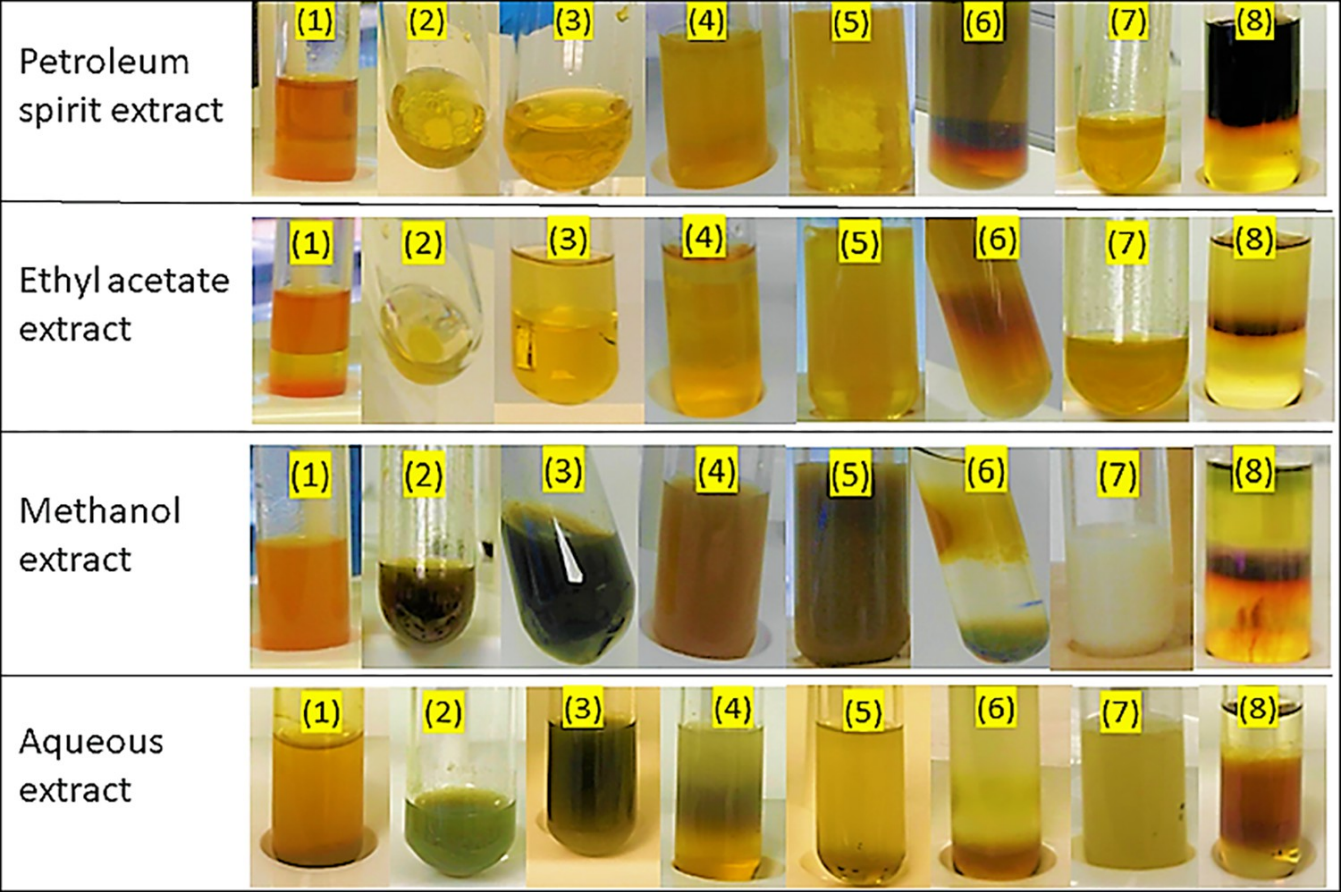
Chemical test of phytochemicals in different extracts of *N*. *sativa* seeds. **(1):** Alkaloids were present in all the extracts showing an orange precipitate, **(2):** Flavonoids were present only in the methanol and aqueous extracts showing an intense green coloration while they were absent in the petroleum spirit and ethyl acetate extracts, **(3):** Phenols were present only in the methanol and aqueous extracts showing deep blue-black colour while they were absent in the petroleum spirit and ethyl acetate extracts, **(4):** Tannins were present only in the methanol and aqueous extracts showing brownish green/dark green coloured solution while they were absent in the petroleum spirit and ethyl acetate extracts, **(5):** Cardiac glycosides were only present in the methanol extract showing blue colour in the acetic acid layer while they were absent in petroleum spirit, ethyl acetate and aqueous extracts, **(6):** Steroids were present in all the extracts showing a reddish-brown coloration, **(7):** Saponins were only present in the aqueous extract showing a stable layer of foam while absent in petroleum spirit, ethyl acetate and methanol extracts, and **(8):** Terpenoids were present in all extracts showing a reddish-brown colour.

The phytochemical screening was achieved through biochemical testing on different extracts (petroleum spirit, ethyl acetate, methanol, and aqueous extracts) of *N*. *sativa* seeds to detect the presence of major natural or medicinally active components. This qualitative screening test showed different results in different extracts of *N*. *sativa* seeds as shown in Fig 1 and Table 1. A significant indication about the presence of alkaloids, steroids and terpenoids was shown in all the extracts of *N*. *sativa* seeds. Other phytochemicals such as flavonoids, phenols, and tannins were only detected in methanol and aqueous extracts. Cardiac glycosides were only detected in methanol extract whereas saponins were only detected in the aqueous extract. Methanol and aqueous extracts of *N*. *sativa* were found to contain phytochemicals in a large amount compared to other extracts.

**Table 1:** Results of phytochemical screening of petroleum spirit, ethyl acetate, methanol, and aqueous extracts of *N*. *sativa* seeds.

S. No.	Name of the phytochemical	Presence (+) and absence (-) in different extracts				Observation
		Petroleum spirit extract	Ethyl acetate extract	Methanol extract	Aqueous extract	
1	Alkaloids	+	+	+	+	Orange precipitate
2	Flavonoids	-	-	+	+	Intense green coloration
3	Phenols	-	-	+	+	Deep blue-black colour
4	Tannins	-	-	+	+	Brownish green/dark green colour
5	Cardiac Glycosides	-	-	+	-	Blue colour in the acetic acid layer
6	Steroids	+	+	+	+	Reddish-brown coloration
7	Saponins	-	-	-	+	Stable layer of foam
8	Terpenoids	+	+	+	+	Reddish-brown colour

The results of the present study showed that *N*. *sativa* seeds are great reservoirs of medicinally active ingredients (phytochemicals) and they are consistent with earlier phytochemical screening studies done on different solvent extracts and oil of *N*. *sativa* seeds which also reported that *N*. *sativa* seeds or extracts and oil are rich in the above-mentioned phytoconstituents [1, 31]. Bioactive constituents such as alkaloids, flavonoids, phenols, tannins and terpenoids are known to elicit broad antimicrobial responses against bacteria, fungi, viruses and parasites [3, 17, 32, 33]. A number of studies documented that these phytochemicals also possess many pharmacological properties, including but not limited to anticancer, antioxidant, anti-inflammatory, cytotoxicity, anti-diarrheal, anti-hemostatic, anti-hemorrhoidal, anti-apoptosis, anti-aging, and growth regulation [31, 34]. Cardiac glycosides possess antihypertensive properties whereas, steroids can act as antibacterial agents and their association with compounds such as sex hormones is significant. As *N*. *sativa* seeds possess these important phytochemical constituents to which their pharmacological activities are ascribed [1, 2, 14, 35], this signifies their potential use as medicine against microbial infections.

### Quantification of phytochemicals

**Fig 2:**
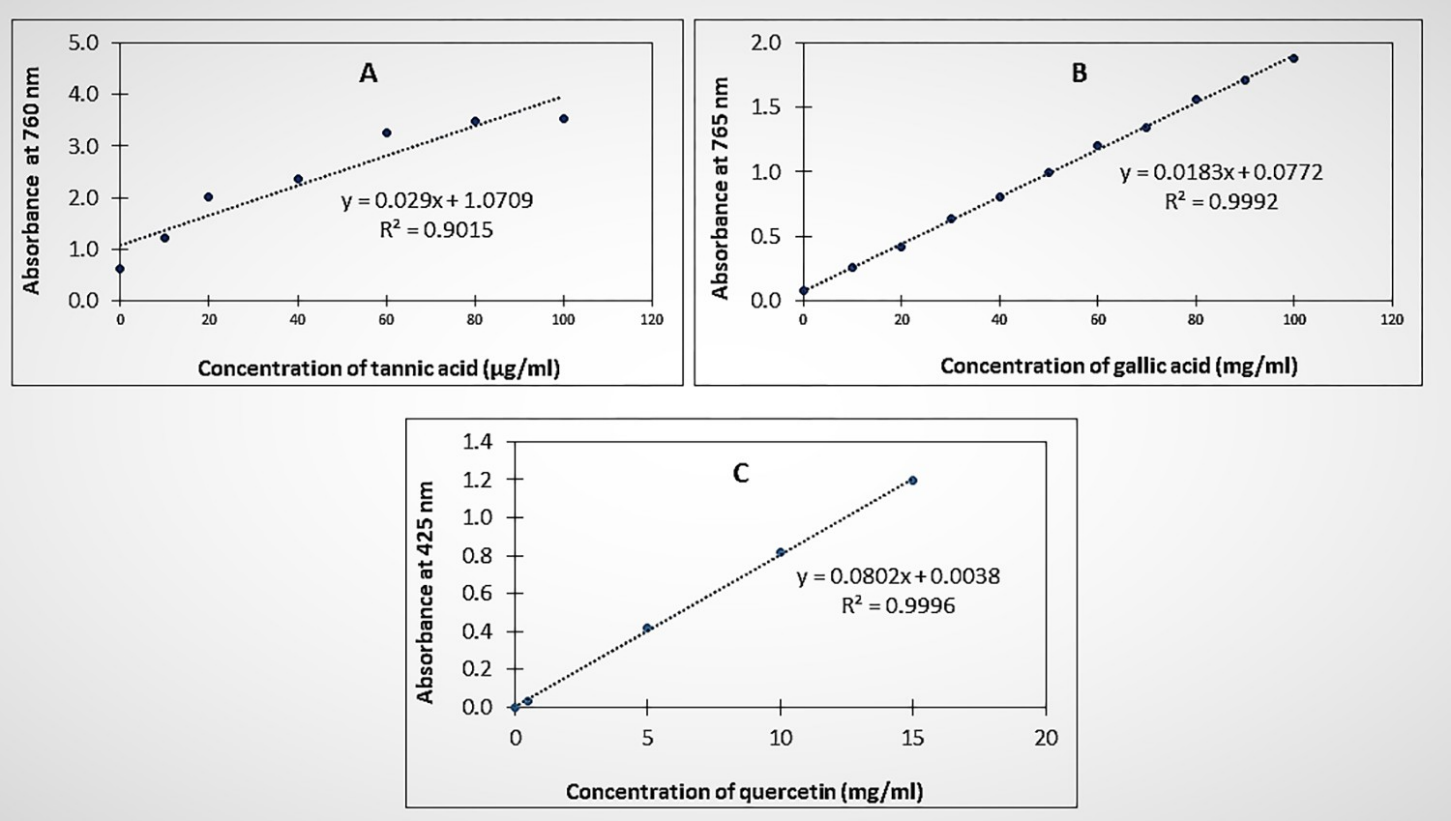
Standard calibration curves for the quantification of phytochemicals in *N*. *sativa* seeds extracts. **(A):** Calibration curve of tannic acid for the determination of total tannin content in aqueous and methanol extracts of *N*. *sativa* seeds. **(B):** Standard calibration curve of gallic acid for the determination of total phenolic content in aqueous and methanol extracts of *N*. *sativa* seeds. **(C):** Standard calibration curve of quercetin for the determination of total flavonoid content in aqueous and methanol extracts of *N*. *sativa* seeds.

The TTC of extracts was calculated in terms of tannic acid equivalents (TAE) by using the standardized linear equation (Fig 2A). The correlation coefficient (R^2^) for the tannic acid calibration was of 0.9015. The tannin content in both aqueous and methanol extract of *N*. *sativa* seeds was 81.7 ± 0.9 μg TAE/g (Table 2). The means of total tannin content (*μ*g TAE/g) in *N*. *sativa* seeds (NSS) extracts obtained by maceration with water and methanol) were not statistically different (F-ration: 2.14 x 10^−5^, p > 0.05). There was also no noticeable difference in the TTC determined in aqueous and methanol extracts. Both solvents were, therefore, best in extracting tannins.

**Table 2:** Content of phytochemicals in the extracts of *N*. *sativa* seeds (mean ± SD; n = 3).

Extract of *N*. *sativa* seeds	Total tannin content (μg TAE/g)	Total phenolic content (mg GAE/g)	Total flavonoid content (mg QE/g)
Aqueous extract	81.7 ± 0.9	71.6 ± 0.0_3_	7.8 ± 0.1
Methanol extract	81.7 ± 0.9	76.5 ± 0.2	8.34 ± 0.0_3_

The total phenolic content (TPC) calculated as the gallic acid equivalents (GAE) from the linear calibration equation of gallic acid (R^2^ = 0.999) (Fig 2B), was of 71.6 ± 0.0_3_ mg GAE/g and 76.5 ± 0.2 mg GAE/g in aqueous and methanol extracts, respectively (Table 2). The means phenolic content of NSS in both solvents appeared to be statistically different (F-ration = 1 528.76, p <0.05). This demonstrated that the TPC varied according to the solvent used with methanol being the best at extracting phenolic content.

The linear equation for quecertin calibration curve (R^2^ = 1.000) was used as a reference to determine the total flavonoid content (TFC) in NSS (Fig 2C). The average TFC (Table 2) obtained by maceration process in methanol (8.34 ± 0.0_3_ mg QE/g) and aqueous (7.8 ± 0.1 mg QE/g) extracts of NSS were significantly different results (F-ratio = 223.28, p<0.05).

These findings demonstrated that *N*. *sativa* seeds are rich in tannin, phenolic and flavonoid contents and concurred with similar studies [1, 4, 14]. In comparison to previous studies, TTC obtained in both aqueous and methanol of NSS (81.7 ± 0.9 μg TAE/g) was lower compared to 754 ± 15 and 1 370 ± 15 mg of catechin equivalent (CE) per g of the sample reported by Aumeeruddy et al. [1]. The amount of phenols in extracts of *N*. *sativa* seeds reported by Saleh et al. [11] was 21 mg/ml in aqueous and 39 mg/ml in methanol extract and it was found to be lower than the content determined in the present study. Varied TPC in different samples of *N*. *sativa* seeds has been documented and thus, the results reported by these authors concur with the present study by demonstrating that *N*. *sativa* seeds are rich in TPC. On the other hand, more amount of flavonoids were found in the methanol extract than in the aqueous extract. Saleh et al. [11] also came up with same observation although their reported TFC was lower than in the present study.

It is well established that methanol (an organic solvent) can extract most active components such as polyphenol or flavonoid from medicinal plant parts than water despite its lower polarity than water [27]. As stated earlier, slight variations were observed in qualitative phytochemical screening results and in the quantitative phytochemical compositions (TTC, TPC and TFC) of *N*. *sativa* seeds. These variations can be attributed to the differences associated with the climatic conditions of the country of origin in which the sample seeds were grown, stage of maturity and due to the analytical techniques and the types of extractants employed [1, 14].

### Thin Layer Chromatographic (TLC) profiling of methanol extract of *N*. *sativa* seeds

TLC studies were performed on methanol extract using two mobile phases which are known to have a good resolution and where more spots were detected [3]. Mobile phase I contained petroleum spirit (PS): ethyl acetate (EA): methanol (ME) in the ratio 2:3:5 and Mobile phase II consisted of Petroleum spirit: ethyl acetate: methanol (6:2:2). The methanol extract was preferred for TLC profiling since it is considered the best solvent for extracting secondary metabolites or phytochemicals than other extractants (petroleum spirit, ethyl acetate and water) according to Sivanandham and Institutions [27] and also as consistently evidenced by both qualitative and quantitative analysis done in the present study.

The TLC profiling of the methanol extract of *N*. *sativa* seeds yielded positive results directing toward the presence of several phytochemicals (S1 Fig and S1 Table). Rf values of 0.03, 0.28, 0.81, 0.91 and 0.14, 0.53, 0.93 obtained with mobile phase I and II, respectively are in agreement with those presented by Mengesha Yessuf [3]. The variations in Rf values of phytochemicals gives crucial ideas in understanding their polarity. They are also known to aid when selecting an appropriate solvent for the separation of these phytochemicals (by using the chromatography and appropriate spectroscopic techniques) from medicinal plant extracts [3, 36, 37]. In this study, more spots were observed in the more polar mobile phase I (S1 Fig and S1 Table) which implies that the separation of phytochemicals from the methanol extract of NSS can be best achieved by using solvents of increasing polarity.

### FT-IR analysis of NSSO

**Fig 3:**
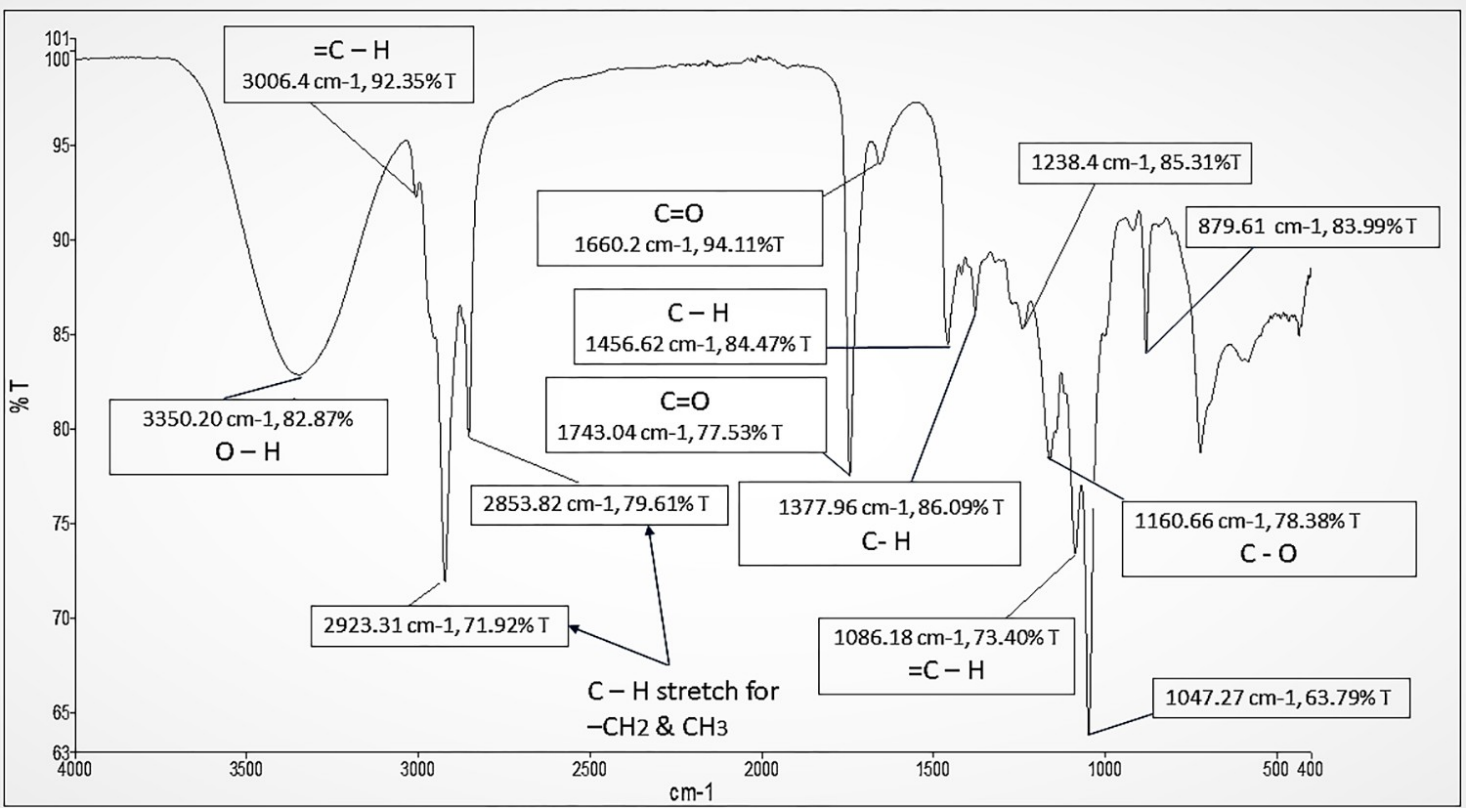
FT-IR spectrum of *N*. *sativa* seeds oil scanned at 4000–400 cm−1. The spectrum illustrates the existence of a variety of peaks at specific wavenumbers that correspond to crucial functional groups that are present in *N*. *sativa* seeds oil extracted using absolute ethanol.

The FT-IR spectrum of *N*. *sativa* seeds oil (NSSO) extracted using absolute ethanol was scanned at the mid infrared region of 400 to 4000 cm^-1^ and a resolution of 4 cm^-1^ was used in order to enhance the sharpness of the peaks (Fig 3). A broad absorption peak at 3350.20 cm^-1^ can be attributed to an O-H functional group which is associated with alcohols and phenols while the weak absorption at 3006.4 cm^-1^ was related to cis = C-H stretching of the vinyl group. In addition, two consecutive intense peaks at 2923.31 cm^-1^ and 2853.82 cm^-1^ were assigned to C-H stretching of an aliphatic group, and it implies the presence of methyl and isopropyl components. Another strong C = O stretching band is present at 1743.04 cm^-1^ which was assigned to oxygen-containing groups such as esters in general. Furthermore, the absorption band shown at 1660.2 cm^-1^ was assigned to the C = O stretching of oxygen-containing constituents particularly in the case where the resonance frequency effect of the carbonyl group is lowered. Another two consecutive peaks were observed at 1456.62 cm^-1^ and 1377.96 cm^-1^. Correspondingly, they were assigned to C-H bending mode (scissoring) and methyl rock. Lastly, a weak peak obtained at 1160.66 cm^-1^ and a band at 1086.18 cm^-1^ were obtained, and they indicated the presence of C-O group and = C-H bending group, respectively.

These findings are in concordance with those reported in previous studies [13, 38]. The identified functional groups imply the presence of main phytochemicals (such as major phenolic compounds, flavonoids and terpenoids) or many therapeutically active components such as thymoquinone in NSSO extracted using absolute ethanol. Here, it is noteworthy that a number of these phytoconstituents of *N*. *sativa* seeds oil in association with their functional groups have been noted for their antimicrobial activity, and they also account for other medicinal properties such as anticancer, hepatoprotective, antioxidant and anti-inflammatory activities [2, 12, 13, 33, 39].

### GC-MS analysis of *N*. *sativa* seeds

**Fig 4:**
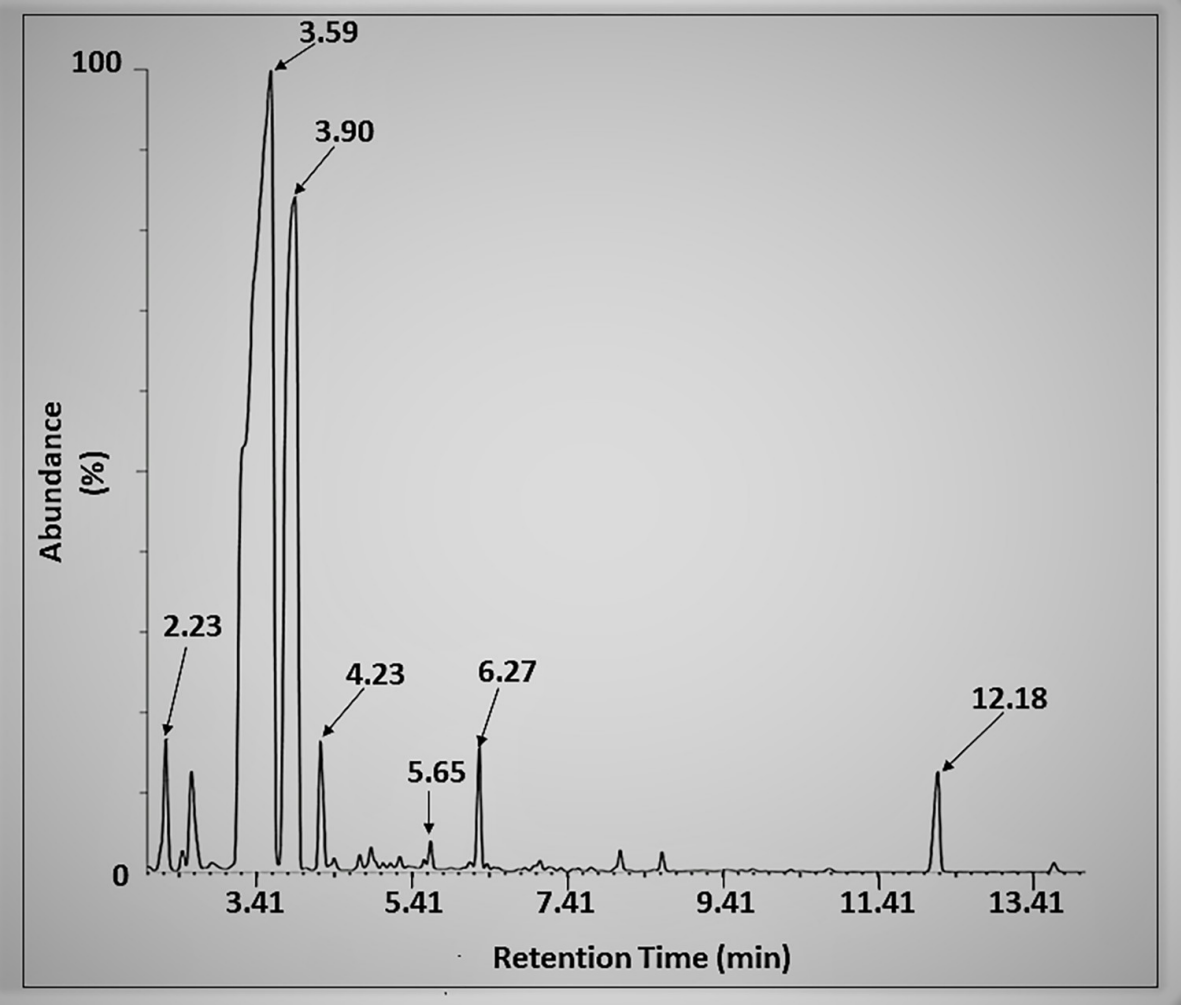
GC-MS chromatogram. Shows NSSO extracted using hexane with 7 major peaks at specific retention times (RT) that correspond to different components i.e., toluene (2.23 min), 4-Pyridineethanesulfonic acid (3.59 min), propanenitrile, 3-chloro (3.90 min), N-(2-Hydroxyethyl)-N-methylaniline (4. 23 min), decane (5.65 min), m-cymene (6.27 min) and allopurinol (12.18 min), respectively.

**Fig 5:**
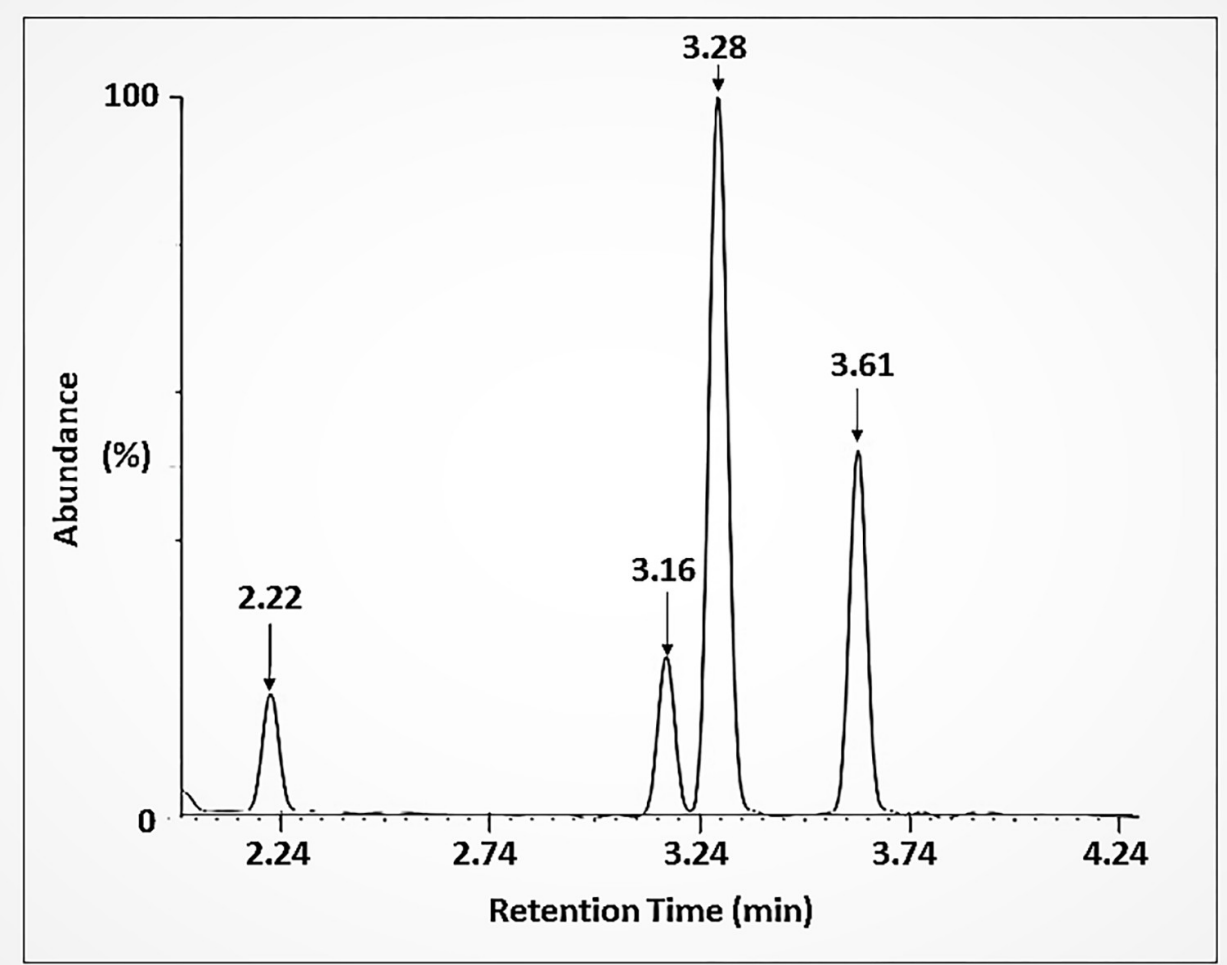
GC-MS chromatogram. Shows methanol extract of NSS with 4 major peaks at specific retention times (RT) that correspond to different components i.e., ethylenimine (2.22 min), ethylbenzene (3.16 min), 1;2-Diphenylamine (3.28 min) and o-xylene (3.61 min), respectively.

The GC-MS chromatogram of NSSO extracted with hexane showed the presence of 7 major peaks (Fig 4) which were of toluene (2.23 min), 4-Pyridineethanesulfonic acid (3.59 min), propanenitrile-3-chloro (3.90 min), N-(2-Hydroxyethyl)-N-methylaniline (4.23 min), decane (5.65 min), m-cymene (6.27 min) and allopurinol (12.18 min). On the other hand, the analysis of the methanol extract showed 4 major peaks and they were identified as ethylenimine (2.22 min), ethylbenzene (3.16 min), 1,2-Diphenylethylamine (3.28 min) and o-xylene (3.61 min) as shown in Fig 5 and Table 3, respectively.

**Table 3:** Major chemical constituents identified in *N*. *sativa* seeds extracts (oil extracted with hexane and methanol extract) and their known medicinal applications or bioactivity.

sample	Constituents	RT (min)	Applications/ bioactivity
NSS oil extracted using hexane	1. Toluene	2.23	• Used to produce cleaning agents/solvents [44, 45]
	2. 4-Pyridineethanesulfonic acid	3.59	Unknown
	3. Propanenitrile, 3-chloro-	3.90	Unknown
	4. N-(2-Hydroxyethyl)-N-methylaniline	4.23	Unknown
	5. Decane	5.65	• Solvent used in organic synthesis as well as in paper and rubber industries [46]
	6. m-cymene	6.27	• Insecticidal and repellant activities [47] • Antimicrobial agent and a flavoring agent for food when isomerized to p-cymene [48]
	7. Allopurinol	12.18	• Xanthine oxidase inhibitor [49]/antigout agent • Radical scavenger [50] • Anti-psoriatic activity and treat kidney stones [51, 52]
Methanol extract	1. Ethylenimine	2.22	• An anticancer agent with physiological effects similar to nitrogen mustards when derivatized [53] • Raw material for cosmetics, ion exchange resins, colloid flocculants and surfactants processing [54]
	2. Ethylbenzene	3.16	• Intermediate for the manufacture of styrene monomer which is a precursor for food containers [55]
	3. 1,2-Diphenylethylamine	3.28	• Possess opioid analgesic effects when derivatized to Lefetamine [56] • Its precursors can be appetite suppressants [57]
	4. O-xylene	3.61	Antioxidant, antimicrobial, and antifungal properties [41]

Of the 7 compounds identified from the hexane extract, only two compounds (toluene and m-cymene) are known to exhibit antimicrobial activity [40], whereas only one compound (o-xylene) from those identified in the methanol extract has been reported to have good antibacterial activity [41]. Based on the above findings, it can be inferred that *N*. *sativa* seeds extracts can be possible sources of antimicrobial agents. In addition to antimicrobial activity, it is also worth noting that the analyzed extracts contained chemical constituents that are also of other medicinal values with a wide range of clinical and industrial applications.

The obtained GC-MS data are consistent with findings from similar studies [11, 12, 41, 42] which reported almost quite identical qualitative composition of different active components in the plant extracts. These studies reported o-cymene and p-cymene (as the main components) in different extracts of *N*. *sativa* seeds, and they mirrored their isomer (m-cymene) which is detected in the current study in the hexane extract. It has also been reported that n-decane is among the main components of *N*. *sativa* seeds volatile oil and it mirrored its isomer (decane) which is detected in the named extract under study [39]. In another study done by François et al. [32] based on other medicinal plants (commiphora species), decane and o-xylene have been detected in extracts using the GC-MS and these results agreed with the findings of the current study. Previous pharmacological studies done on *N*. *sativa* seeds and their extracts well attributed them as food flavoring agents and appetite stimulants or suppressants. They also confirmed that *N*. *sativa* seeds and their extracts impart anti-psoriatic, anticancer, analgesic effects, antioxidant, and defense by scavenging and antifungal activity, just to mention a few bioactivities that mirrored the results of the current study. In addition, *N*. *sativa* seeds have been used since ancient time as the richest source of nutraceuticals, pharmaceutical intermediates, and chemical entities of traditional or modern synthetic drugs due to their diversity in chemical constituents [1, 10, 14, 35, 43]. The results in Table 4 clearly emphasize the reasons *N*. *sativa* seeds have been used for quite a long time in traditional folk medicine based on the activity of their active ingredients.

**Table 4:** Zones of inhibition of different preparations of *N*. *sativa* seeds, positive treatment, and negative treatment against pathogenic bacteria.

		Zone of inhibition (mm)			
Bacteria →		*E*. *coli*	*P*. *aeruginosa*	*S*. *aureus*	*B*. *subtilis*
*N*. *sativa* seeds preparations (10 μl/disc)	Oil (extracted using absolute ethanol) undiluted	7.99 ± 0.01	6.58 ± 0.09	11.62 ± 2.67	12.33 ± 0.82
	Oil (extracted using absolute ethanol) diluted (1:1)	-	7.09 ± 1.54	9.47 ± 0.71	8.91 ± 0.60
	Oil (extracted with hexane) undiluted	8.92 ± 0.54	6.86 ± 0.12	13.06 ± 1.04	12.83 ± 2.27
	Oil (extracted with hexane) diluted (1:1)	7.02 ± 0.85	6.53 ± 0.36	10.03 ± 0.73	9.10 ± 1.47
	Methanol extract undiluted	7.85 ± 0.60	7.94 ± 0.11	7.57 ± 0.79	7.77 ± 1.14
	Methanol extract diluted (1:1)	7.72 ± 0.89	6.64 ± 1.00	-	6.76 ± 0.25
	Aqueous extract undiluted	-	-	-	8.12 ± 0.49
	Aqueous extract diluted (1:1)	-	-	-	6.34 ± 0.98
Positive treatment	Streptomycin (S) (Standard antibiotic)	25.82 ± 1.16	30.21 ± 0.15	15.31 ± 1.41	27.05 ± 1.76
Negative treatment	Double distilled water	-	-	-	-

Interpretation of results:—(-) implies no zone of inhibition observed

The values are expressed as mean ±SD of the three replicates

Significant at p<0.05

The GC-MS analysis results raised a distinct possibility that antimicrobial agents can be harnessed from *N*. *sativa* seeds (a natural source) to develop new intervention methods (such as designing antibiotics or discovering new antimicrobial drugs) to halt the continuous rise of drug resisting pathogenic bacterial strains or cure life-threatening diseases. However, there is a need to confirm the presence of these “newly” identified compounds in *N*. *sativa* seeds through appropriate approaches. It was reported that the geographical origin in which the sample seeds were grown, the extraction and processing techniques affect the chemical composition of the extracts or account for variation from literature in addition to other reasons mentioned earlier [1, 12, 14].

### Antimicrobial activity of *N*. *sativa* seeds extract

The antibacterial activity of *N*. *sativa* seeds was investigated against some of the common-known pathogenic bacteria (i.e., *E*. *coli*, *P*. *aeruginosa*, *S*. *aureus* and *B*. *subtilis*). The observed zones of inhibition from different extracts were measured and confirmed using streptomycin (standard antibiotic) as a positive control, and distilled water as a negative control (Table 4). Different extracts and/or oil of *N*. *sativa* seeds showed complex antimicrobial activities against the pathogenic bacteria strains with different efficacy. The inhibitory activity of streptomycin (the best positive control) against the tested bacteria showed the following order: *P*. *aeruginosa* (30.21 ± 0.15) > *B*. *subtilis* (27.05 ± 1.76) > *E*. *coli* (25.82 ± 1.16) > *S*. *aureus* (15.31 ± 1.41). Cephalothin (KF), ampicillin (AMP) and penicillin (P) were also used as positive controls however, their inhibitory activity were not consistent against all the bacteria. Hence, their inhibitory effects were not taken into consideration. In addition, no antibacterial or inhibitory activity was attributed to the negative treatment for all the tested pathogenic bacterial strains.

**Fig 6:**
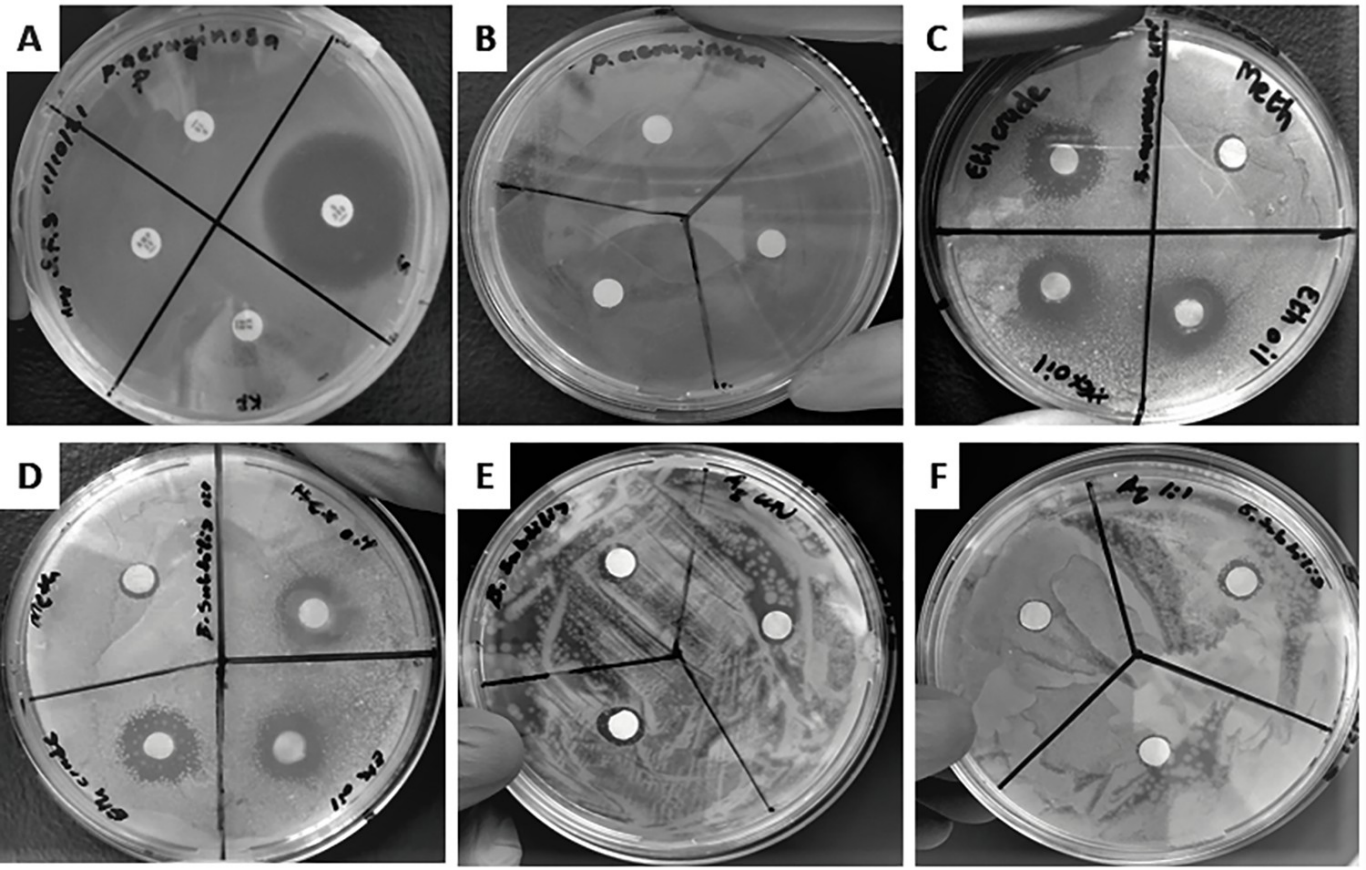
Inhibitory effects of different preparations/extracts of *N*. *sativa* seeds, positive treatment, and negative treatment against pathogenic bacterial strains. **(A):** Inhibitory activity of streptomycin (S) against *P*. *aeruginosa*. **(B):** Negative treatment using double distilled water no inhibitory activity was observed for all the tested pathogenic bacterial strains (i.e., *P*. *aeruginosa*). **(C):** Inhibitory activity of undiluted extracts of *N*. *sativa* seeds (oil extracted using absolute ethanol, oil extracted using hexane and methanol extract) against *S*. *aureus* where absolute ethanol had the highest zone of inhibition followed by oil extracted using absolute ethanol and oil extracted using hexane while methanol extract was the least potent. **(D):** *B*. *subtilis*, all diluted extracts were active with reduced efficacy as opposed to that of the undiluted ones. **(E):** Inhibitory activity of the undiluted aqueous extract against *B*. *subtilis*. **(F):** Inhibitory activity of the diluted aqueous extract with reduced efficacy compared to the undiluted aqueous extract.

Different solvent extracts and/or preparations of *N*. *sativa* seeds showed broad spectrum and significantly different activity, inhibiting both gram-negative (*E*. *coli* and *P*. *aeruginosa*) and gram-positive (*S*. *aureus* and *B*. *subtilis*) pathogenic bacteria in the disc diffusion method (F-ratio = 7.22, p<0.05) as shown in Fig 6. For the antimicrobial activity of undiluted extracts of *N*. *sativa seeds* performed using the disc diffusion assay on nutrient agar plates, the inhibitory effect of hexane oil extract against the bacteria followed the order: *B*. *subtilis* > *S*. *aureus* > *E*. *coli* > *P*. *aeruginosa*. A similar trend was observed in the zones of inhibition for the oil extracted using absolute ethanol but *P*. *aeruginosa* was the most susceptible microbe than *E*. *coli*. The undiluted methanol extract was more effective against both gram-negative and gram-positive bacteria almost to the same extend and its inhibitory effects towards the bacteria followed the order: *P*. *aeruginosa* > *E*. *coli* > *B*. *subtilis* > *S*. *aureus* unlike for other extracts. This can be attributed to better extraction of phytochemicals as demonstrated by qualitative and quantitative analysis like in the case of methanol and to the differences in the chemical composition of different solvent extracts. The aqueous extract only showed antibacterial activity against *B*. *subtilis*.

These observations are in agreement with a study done on *Tagetes erecta* flowers by Padalia and Chanda [15] and similar works by Sivanandham and Institutions, and Shikwambi et al. [27, 28] who showed poor inhibitory activity of the aqueous extracts than organic solvent extracts in which they could not inhibit most of the tested microorganisms. They concluded that most water-soluble phytochemicals such as flavonoids may lack antimicrobial significance and phenolic compounds may only be significant as antioxidants. In addition, it was indicated that even though water is a universal solvent that is widely used by traditional healers, organic solvents such as methanol, ethanol and hexane have been known to extract more of the bioactive constituents or antibacterial agents and hence, they give consistent antimicrobial activity than water. It was observed in the current study that most extracts and/or oil with exception to the methanol extract had large zones of inhibition against gram-positive strains as opposed to gram-negative and thus, they were more potent towards the gram-positive bacteria. These results concurred with findings of similar studies by Mohammed et al. and Saleh et al. [11, 13] which reported that gram-negative bacterial strains have an outer cell membrane inside the cell wall which can act as a permeability barrier. This can minimize the uptake of antimicrobial compounds such as polyphenols by the bacterial cell and hence, resulting into the extracts being less effective on gram-negative bacteria compared to their counterpart.

Evaluation of the antimicrobial activity of undiluted extracts and oil of *N*. *sativa seeds* was supplemented by an additional disc diffusion test on nutrient agar plates using the same extracts and/or oil but diluted 1:1 in ethylene glycol. The diluted extracts and oil were also active against the tested bacterial strains with different efficacy as exhibited by their corresponding undiluted extracts of *N*. *sativa seeds*, but it was noted that most of them had lower antimicrobial activity than that of the undiluted ones (Fig 6). On the contrary, *E*. *coli* and *S*. *aureus* remained unaffected by the diluted oil extracted with absolute ethanol and diluted methanol extract, respectively. In this study, it is speculated that these diluted extracts may be immiscible in the aqueous microbial growth medium resulting in lower or no inhibitory activity as opposed to the activity of undiluted extracts. Taken together these findings support the notion that *N*. *sativa* seeds and their extracts possess significant antibacterial activity and they are consistent with those in a similar study done by Saleh et al. [11], although with different efficacy. In this supplementary disc diffusion assay, ethylene glycol was used to serve as a mean to deliver or control the release of antibacterial agents (bioactive compounds) from the extracts of *N*. *sativa* seeds to leverage its benefits and provide an effective therapeutic treatment [58]. The use of ethylene glycol to control the release of antibacterial agents from *N*. *sativa* extracts allowed the preparations to be directly pipetted onto their respective 5 mm discs without spreading all over the plates and this would reduce the chances for the zones of inhibitions to overlap.

The antimicrobial activity induced by various extracts and/or oil of *N*. *sativa* seeds against pathogenic bacteria was linked to the antimicrobial phytoconstituents present in them as well as to the complex interaction between those components and other components present in the extracts and/or oil of *N*. *sativa* seeds [11, 13]. As evidenced by earlier phytochemical investigations done in this study, methanol and aqueous extracts of *N*. *sativa* seeds contained therapeutically active components such as alkaloids, tannins, phenols and flavonoids or other phytochemicals which have been recognized as antimicrobial compounds and thus, their synergism mechanism against predation by pathogenic microorganisms can serve as a defense [16, 33, 59]. In addition, the antimicrobial activity of the methanol extract may be due to o-xylene as its component identified by GC-MS analysis which has been reported to have good antibacterial activity [41]. In studies of this kind, influences from the existence of some minor unidentified constituents with antimicrobial properties in the extracts cannot be neglected [60]. The FT-IR analysis performed in this study revealed that oil of *N*. *sativa* seeds extracted with absolute ethanol is enriched with different kinds of phytoconstituents whose associated characteristic functional groups (antibacterial features) such as O–H (in phenolic compounds, flavonoids, and alcohols), C–H (for terpenes) and esters (C = O) could be responsible for the inhibition of some parts of the bacterial cell or account for antibacterial activity [13, 33]. The antimicrobial activity of *N*. *sativa* seeds oil extracted using hexane may be attributed to the synergism effect of two of its bioactive compounds identified by GC-MS analysis which can exhibit antimicrobial activity, toluene and m-cymene [44, 45, 48] which need to be confirmed by further studies.

Phytoconstituents in the different extracts and/or oil of *N*. *sativa* seeds can interact with the membranes of the bacteria and eventually cause disruption with the aid of lipophilic products (Fig 6). Concerning the mechanism of inhibition of bacterial growth, the bacterial cell membrane can be forced to break down and release the content of the cell. In general, the bacterial cells die due to such breakdowns which trigger inhibition of respiration and increase membrane fluidity and permeability of both bacterial stains [13]. This study observed that different preparations of *N*. *sativa* seeds have different constituents occurring in varying amount and this could be the reason for the differential inhibitory activity against the bacteria. Literature reports revealed that the antibacterial activity depends on the extraction technique employed used to extract the bioactive phytochemicals from the seeds, the structure of the bioactive components in the extracts and the polarity of the bioactive components in the extracts [17, 25, 39].

Irregular zones of inhibition were observed in this study around the discs (Fig 6) and Khan et al. [17] had reported that it may be due to improper diffusion of the extracts or uneven growth of the bacteria at a particular area of the nutrient agar. Most of these sources of variations (i.e., the type of extractant used, extraction technique employed, the type of pathogenic bacteria strains used and variation due to error) were confirmed by the two-factor ANOVA statistical analysis done on mean values of replication of the zones of inhibition of different extracts and/or oil of *N*. *sativa* seeds and the results revealed that they were highly significant at p<0.05. The antibacterial activity displayed by different preparations of *N*. *sativa* seeds were consistent with both qualitative and quantitative investigations regarding their composition (Tables 1 and 2), and structural characterization by FT-IR and GC-MS techniques (Figs 3–5), verifying that the data of the zones of inhibition were accurate and reliable (Table 4). The standard deviations for all the statistical analysis done in this study were lower, indicating that the values are clustered around the true mean of the set (Tables 2 and 4).

Although this study offers strong insight into the phytochemical compositions and antimicrobial activity of *N*. *sativa* seeds, it had experienced some limitations which are noteworthy. Firstly, methanol was one of the widely used solvent in the laboratory and its use in the separation of phytochemical compounds by using the column chromatography could accelerate its depletion hence, this study only performed TLC analysis of the methanol extract. Secondly, some of the extracts or preparations of *N*. *sativa* seeds were precipitating upon addition to the inoculated plates.

## Conclusion

The characterization of the phytochemical composition and antimicrobial activity of *N*. *sativa* seeds was carried out using different extracts. Medicinally active components of interest from *N*. *sativa* seeds were successfully extracted using different solvents (petroleum spirit, ethyl acetate, methanol, water, and hexane), identified, quantified, and characterized using optimum methods or techniques.

The extraction of the oil from *N*. *sativa* seeds was achieved by Soxhlet extraction method using two extractants namely, absolute ethanol and hexane which are known to be suitable when extracting oil for testing biological activity. The phytochemical screening investigations found most of the major bioactive phytochemicals in different extracts of *N*. *sativa* seeds and this ascertained their medicinal value. Regarding the determination of total phytochemical content, higher total phenolic and flavonoids contents were found in methanol extract compared to the aqueous extract of *N*. *sativa* seeds while, the total tannin content in the two extracts studied was the same. The TLC study provided a reliable experimental basis for further separation of phytochemicals from the methanol extracts of *N*. *sativa* seeds using appropriate solvents and this basis is consistent with a study done by Mengesha Yessuf [3]. On the other hand, the FT-IR analysis of *N*. *sativa* seeds oil extracted using absolute ethanol revealed different functional groups of antimicrobial value and they can also play prominent roles in other biological activities of *N*. *sativa* seeds.

Based on the reports from literature on the bioactivities and/or medicinal applications of compounds detected by GC-MS in this study, oil extracted using hexane and methanol extract of *N*. *sativa* seeds can be exploited to promote the use of *N*. *sativa* seeds as natural curative remedies against predation by pathogens that have been linked with causing diseases in human and this was ascertained by the antimicrobial analysis. The GC-MS results also signify the use of *N*. *sativa* seeds in many pharmaceutical settings due to their diversity in composition however, there is a need to confirm the presence of these newly identified chemical constituents. The phytoconstituents and/or the complex interactions among other constituents present in the extracts and/or oil of *N*. *sativa* seeds may be responsible for their indispensable antimicrobial activity. Therefore, the seeds of *N*. *sativa* can be used as the natural source of antimicrobial agents in the pursuit of searching for new antibiotics against human pathogens as it is evident with the present results.

### Statistical analysis

All experiments were performed in triplicate unless stated otherwise. Statistical analysis of the data was performed using Statistical Software for Social Sciences version (SPSS) version 24. A p value <0.05 was regarded as significant.

## Supporting information

S1 Fig TLC profile of methanol extract of *N*. *sativa* seeds. Plate 1 was exposed to PS: EA: ME (2:3:5) and Plate 2 was exposed to PS: EA: ME (6:2:2).
(PDF)

S1 Table TLC profile of methanol extract of *N*. *sativa* seeds. Retention factor (Rf) values of the extract.
(DOCX)
