# Supplementary figures and images for: Phytochemical characterization and antimicrobial activity of Nigella sativa seeds
Source: PLoS One. 2022 Aug 4;17(8):e0272457. doi: 10.1371/journal.pone.0272457 (PMC9352024; doi:10.1371/journal.pone.0272457)

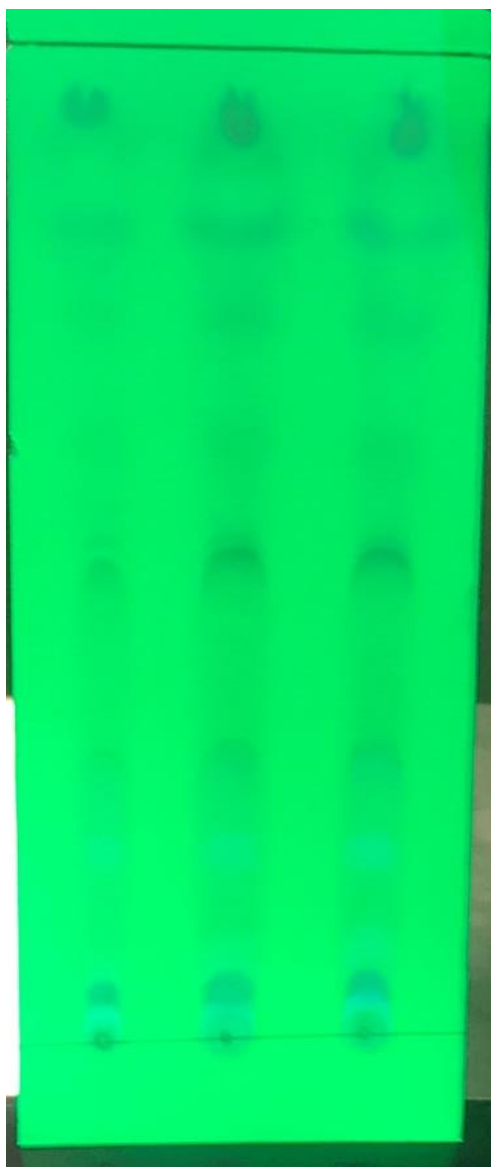

Plate 1 in S1 Fig

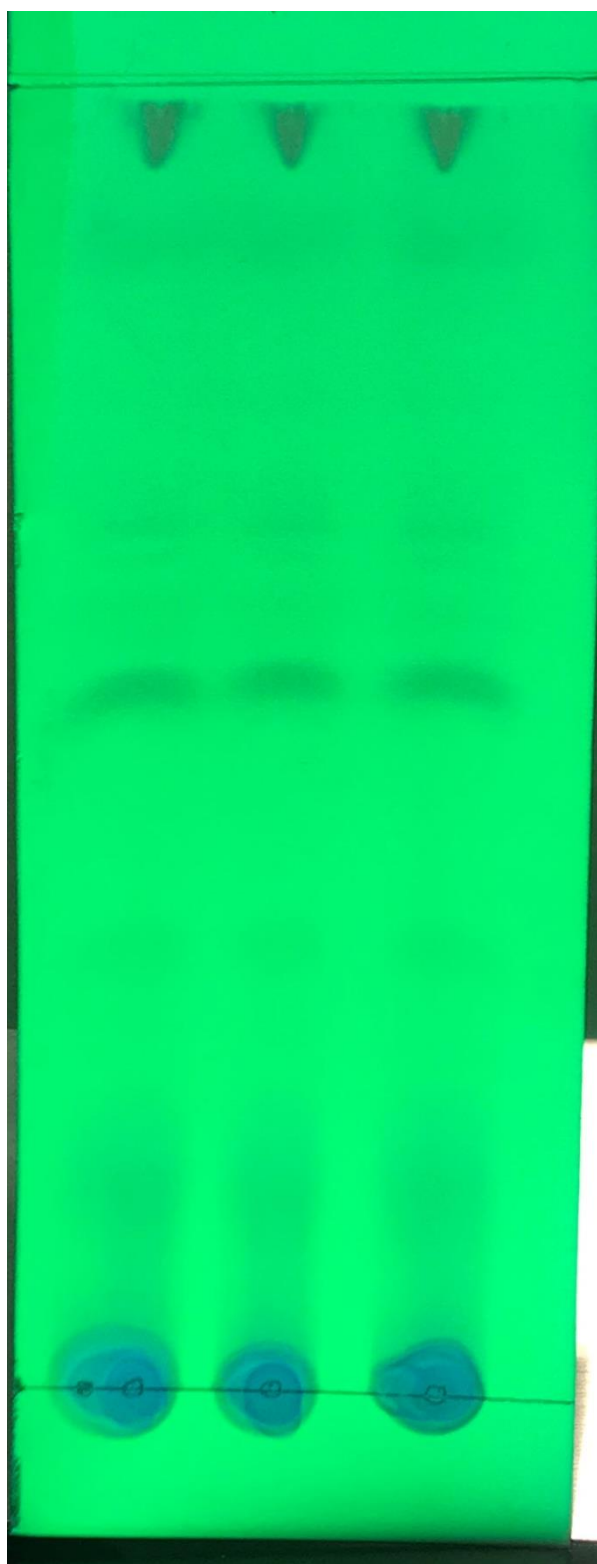

Plate 2 in S1 Fig

Supplement: S1 Fig — Plate 1 was exposed to PS: EA: ME (2:3:5) and Plate 2 was exposed to PS: EA: ME (6:2:2). (PDF) [file pone.0272457.s001.pdf]
