# Supplementary material for: Phytochemical characterization and antimicrobial activity of Nigella sativa seeds
Source: PLoS One. 2022 Aug 4;17(8):e0272457. doi: 10.1371/journal.pone.0272457 (PMC9352024; doi:10.1371/journal.pone.0272457)
Supplement: S1 Table — Retention factor (Rf) values of the extract. (DOCX) [file pone.0272457.s002.docx]

# Supporting information

| No. of TLC plate | Plate 1 | Plate 2 |
| --- | --- | --- |
| Mobile phase | I | II |
| Ratio | 2:3:5 | 6:2:2 |
| Total number of spots visualized | 4 | 3 |
| Rf value | 0.03, 0.28, 0.81, 0.91 | 0.14, 0.53, 0.93 |

**S1 Table. TLC profile of methanol extract of *N. sativa* seeds.** Retention factor (Rf) values of the extract.
